# A commentary on ‘Laparoscopic versus open repeat hepatectomy for recurrent hepatocellular carcinoma: a systematic review and meta-analysis of propensity score-matched cohort studies’

**DOI:** 10.1097/JS9.0000000000000517

**Published:** 2023-05-24

**Authors:** Furui Zhong, Chuanbo Xie, Xuefeng Peng, Hua Yang

**Affiliations:** a Department of General Surgery of Huidong, Zigong Fourth People’s Hospital; b Department of Ultrasound, Zigong Hospital of Woman and Children Healthcare, Zigong, Sichuan Province, People’s Republic of China

*Dear Editor*,

For patients with recurrent hepatocellular carcinoma (RHCC) that can be resected, repeat hepatectomy provides a better long-term prognosis than nonsurgical treatments^1^. Laparoscopic hepatectomy, with its advantages of less blood loss, fewer postoperative complications, and shorter hospital stays compared to the open approach, is considered a safe and feasible method for the treatment of HCC^2^. However, laparoscopic repeat hepatectomy (LRH) for RHCC presents challenges related to intraperitoneal adhesions, changes in residual liver volume, and local anatomical alterations resulting from the initial procedure.

We read with great interest the recent meta-analysis by Xiang *et al.* ^3^, reporting the surgical and oncological outcomes of LRH and open repeat hepatectomy (ORH) for RHCC. The authors included five studies involving a total of 818 patients and concluded that LRH might be a preferable treatment option for RHCC. Most surgical outcomes with LRH, including blood loss, operative time, major complication rate, and length of hospital stay, were superior to those of ORH, with comparable oncological results. Although this study was well-designed and high-quality, several issues warrant further discussion.

First, apart from the five articles selected by the authors, two additional studies^4,5^ were found to meet the inclusion criteria and should be considered for pooled analysis in this systematic review. Second, there were several misinterpretations of the results in this article. Regarding tumor size, the pooled data mean difference (MD)=−0.22 and 95% confidence interval (CI): [−0.45 to −0.01] indicated that the LRH group had a smaller tumor size than that of the ORH group. However, the authors interpreted this as no significant difference between the two groups. Likewise, for operation time, the analysis of MD=66.19 and 95% CI: [5.28–127.10] showed that the operation time in the LRH group was significantly longer than that in the ORH group. Nevertheless, the author mistakenly claimed that the operation time in the LRH group was shorter, leading to the opposite conclusion. Third, it is not appropriate for the authors to directly use a random-effects model for analysis without considering heterogeneity between the studies. Furthermore, it has been suggested that the fixed-effects model should be adopted for meta-analyses that include fewer than five studies^6^. Fourth, the study by Nomi *et al.* ^7^ was published in 2023, but all forest plots were incorrectly labeled as ‘Nomi 2022’. Correcting this minor error is necessary. Finally, it is preferable to use Begg’s and Egger’s tests instead of funnel plots for the data in this study to improve the reliability and accuracy of assessing potential publication bias in the five included studies^8^.

Overall, we offer much appreciation to the authors for their contribution in presenting us with a comprehensive meta-analysis and providing significant insight into the effectiveness of LRH and its potential benefits in RHCC. In the future, we should also aim to accurately identify which subset of patients with RHCC would benefit most from LRH.

## Ethical approval

Not applicable to this study.

## Sources of funding

The comment does not receive any funding.

## Author contribution

F.Z.: study design and writing; C.X. and X.P.: critical review; H.Y.: study supervision.

## Conflicts of interest disclosure

The authors declare that they have no conflicts of interest.

## Research registration unique identifying number (UIN)

1. Name of the registry: not applicable.
2. Unique identifying number or registration ID: not applicable.
3. Hyperlink to your specific registration (must be publicly accessible and will be checked): not applicable.

## Guarantor

Furui Zhong and Hua Yang.
